# Allergic reaction to temporary percutaneous peripheral stimulation leads: A case report

**DOI:** 10.1016/j.inpm.2025.100562

**Published:** 2025-03-08

**Authors:** Amir Soheil Tolebeyan, Kimberly Youngren

**Affiliations:** Center for Pain and Spine, Dartmouth Hitchcock Medical Center, Dartmouth Geisel School of Medicine, USA

Dear Editor,

In the 1960s, Wall and Melzack proposed the gate-control theory, which suggests that non-nociceptive A-beta fibers can suppress nociceptive signals transmitted by A-delta and C-fibers [1]. Although the exact mechanism of percutaneous peripheral nerve stimulation (PNS) is not fully understood, it is believed to work based on the gate control theory by activating A-beta fibers that inhibit pain. This activation enhances the function of inhibitory dorsal neurons in the spinal cord, suppressing the excitatory A-delta and C fibers [1]. Temporary percutaneous peripheral nerve stimulation aims to generate proprioceptive afferent signals over a 60-day treatment period. This process restores the balance of peripheral inputs to the central nervous system and reverses maladaptive changes in central pain processing [2]. Temporary PNS is indicated for use in chronic pain, post-surgical and post-traumatic acute pain, as well as post-traumatic and post-operative pain [3]. However, it could be contraindicated in patients with a history of tape or adhesive allergy, epilepsy, or those who already have an active cardiac implant or deep brain stimulation. Additionally, most of these leads are not MRI-compatible and must be removed before undergoing an MRI study [3].

Although there are limited available studies reporting adverse reactions to temporary PNS, pivotal data indicates that skin rash is the most common side effect of the device, followed by itching at the exit site of the electrodes, granuloma, infection, pain after electrode placement, and lead migration [3]. To our knowledge, this report is the first case of generalized skin rash due to temporary peripheral nerve stimulation lead placement reported in the literature.

We are presenting a 64-year-old woman with a history of L3-S1 fusion, post-laminectomy syndrome with a history of successful spinal cord stimulator implant, sacroiliac joint dysfunction, and hypertension, who underwent percutaneous peripheral nerve stimulation placement using the SPRINT TM, targeting bilateral S2 lateral branch at the level of the foramen for her sacroiliac joint pain. The patient has a history of allergy to adhesive bands (EKG patches cause erythema), naproxen, codeine, and lisinopril.

Approximately 30–60 minutes after discharge, the patient developed a diffuse rash and hives without any other signs of severe allergic reaction. Initially, this was assumed to be due to perioperative antibiotics or anesthetics. However, over the next three weeks, she developed papules across her body, some with oozing (Fig. 1, Fig. 2, Fig. 3, Fig. 4, Fig. 5). There were no accompanying symptoms such as fever, chills, or flushings. The patient took diphenhydramine, which improved but did not fully alleviate her symptoms. Despite removing the adhesive bandage and trying different types of adhesives, there was no improvement in her symptoms.

**Fig. 1:**
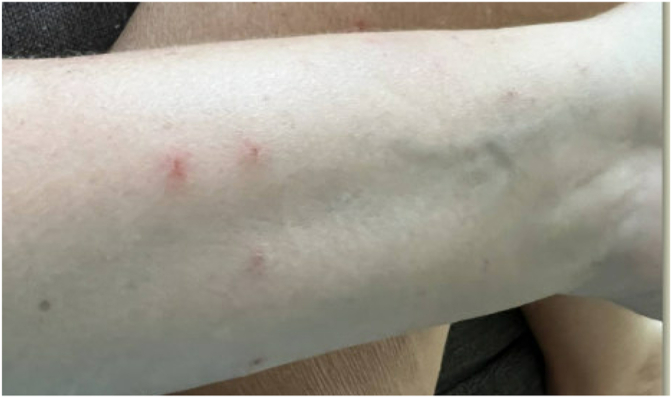
Skin rashes on the patient’s forearm.

**Fig. 2:**
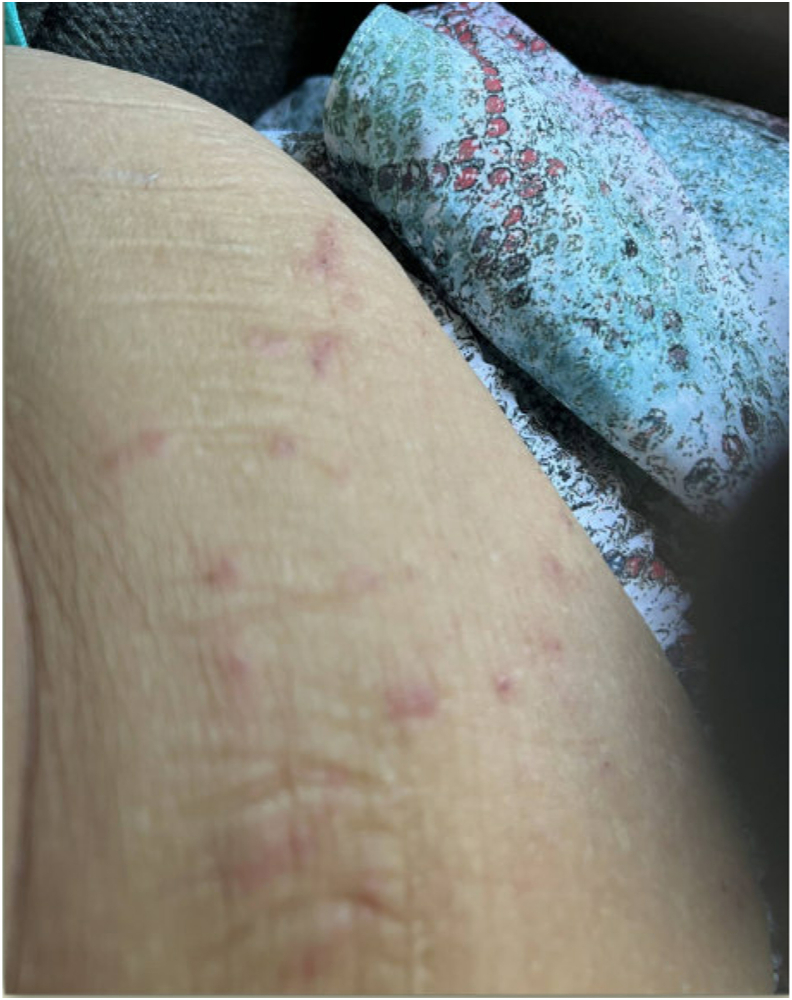
Skin rashes on the patient’s abdominal wall.

**Fig. 3:**
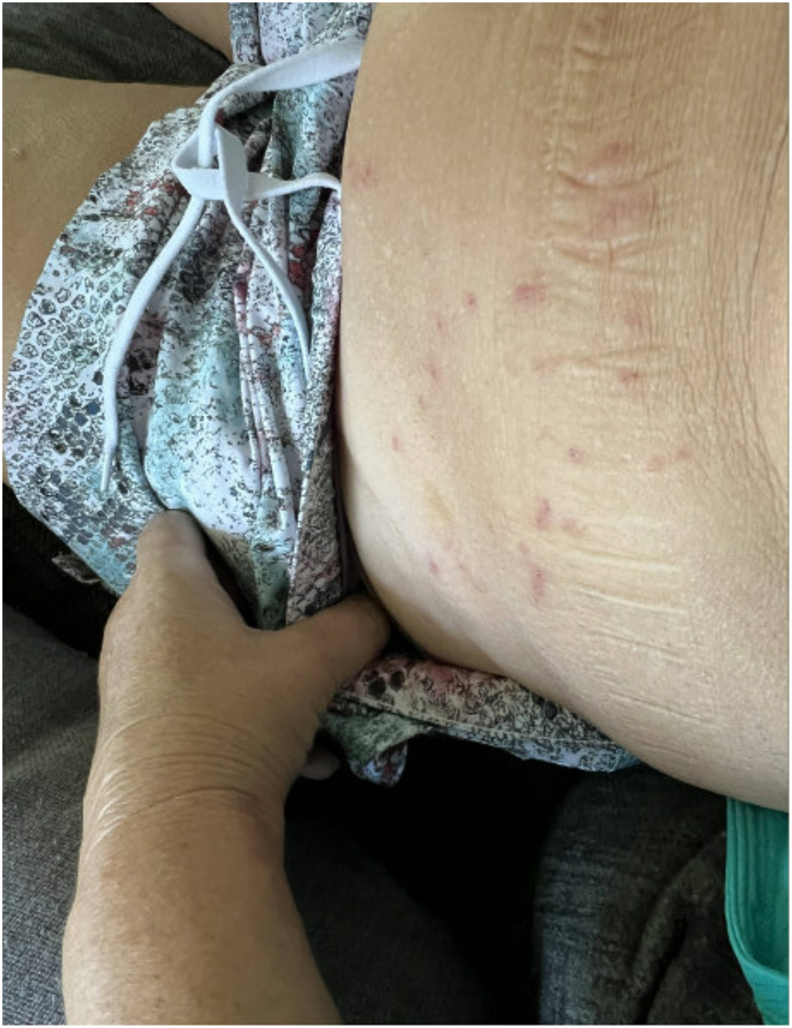
Skin rashes on the patient’s abdominal wall.

**Fig. 4:**
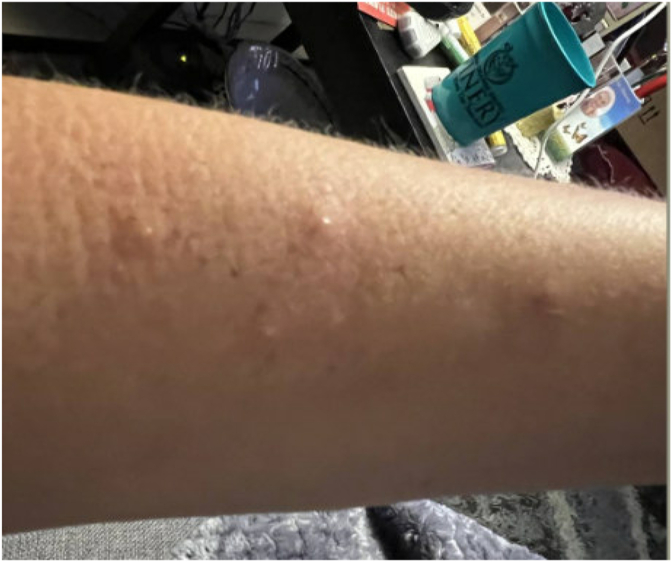
Skin rashes on the patient’s forearm.

**Fig. 5:**
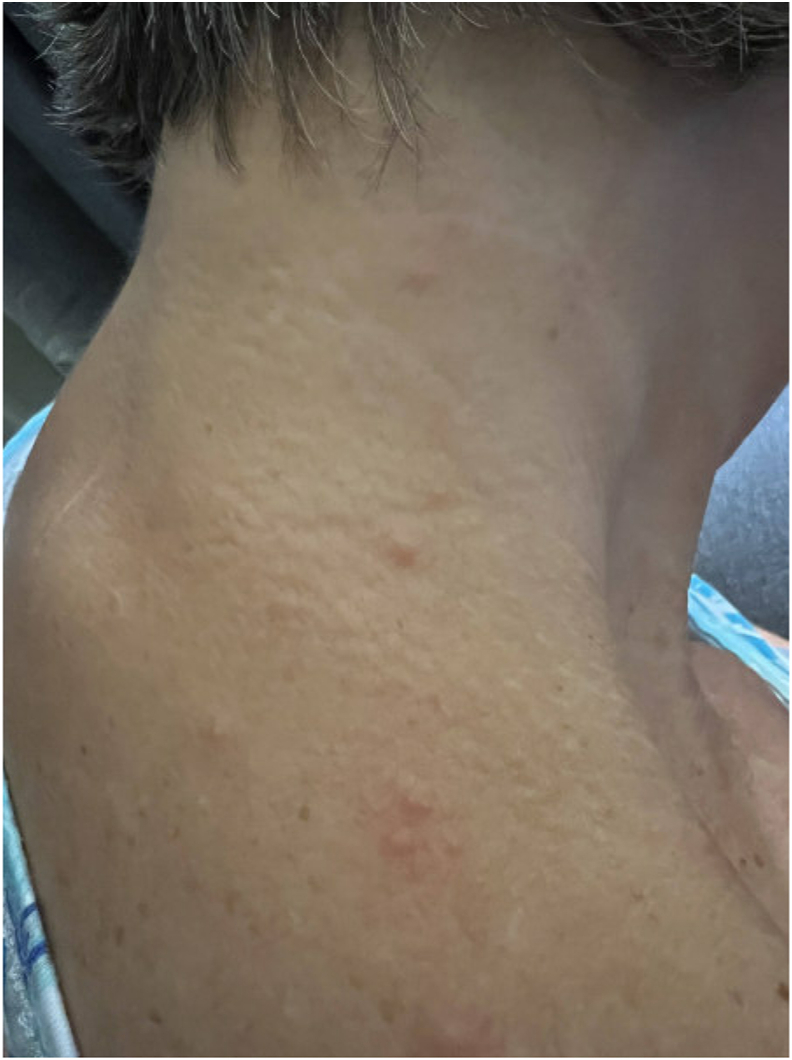
Skin rashes on the patient’s shoulder and neck.

On physical examination, hives and papules were observed on both legs, shoulders, upper back, and neck.

Ultimately, we decided to remove the leads, which were taken out about three weeks after placement despite excellent pain relief from the device due to ongoing rash and pruritis. The patient's symptoms completely resolved within a few days following the lead removal. The patient reported a remote nickel allergy during the follow-up visit. Four months after the lead removal, the patient underwent a bilateral S2 permanent peripheral nerve stimulation implant using Curonix. No allergic reaction developed during the 4 weeks post-operation.

According to the Gell and Coombs system, immunologic reactions are classified into four categories: Type I, Immediate onset, mediated by mast cells and IgE, with/without basophils; Type II, Delayed onset antibody-mediated cell destruction; Type III, Delayed onset due to the drug immune complex deposition and complement activation; and Type IV, Delayed onset T cell-mediated [4].

Both Type I and IV allergic reactions could occur due to metals. Type I typically induces urticaria-type reactions. However, Type IV hypersensitivity reaction is known as the most common cause of allergic contact dermatitis caused by metals [5]. With an estimated prevalence of 20 %, nickel takes the lead in this category, followed by 1%–3% due to other metals, such as cobalt and chromium [5].

Vasoactive mediators released by mast cells may cause rash, pruritis, wheezing, diarrhea, and anaphylaxis in type I hypersensitivity [4]. Based on the administration route, symptoms' onset time varies: seconds to minutes for intravenously administrated medications, 3–30 minutes for orally administrated medications, and 3–60 minutes if medications are taken orally [4].

A previous exposure is usually required for IgE-mediated reactions. However, sensitization resulting from exposure to a cross-reactive compound in the past could still cause this type of hypersensitivity reaction [4]. Unlike other types of hypersensitivity, type IV is triggered by T cell activation and, in some cases, neutrophils, macrophages, and eosinophils. The symptoms of type IV usually start within 48–72 hours following exposure. Examples of type IV include contact dermatitis, maculopapular eruptions, Symmetrical drug-related intertriginous and flexural exanthem, Acute generalized exanthematous pustulosis, Stevens-Johnson syndrome, and toxic epidermal necrolysis, drug fever, Drug-induced hypersensitivity syndrome [4].

Univers et al., in 2018, reported a case of systemic hypersensitivity reaction to an endovascular stainless steel stent. Given the history of nickel allergy, a stainless steel stent was chosen to be implanted in the right iliac artery. However, the patient presented with a systemic eczematous rash that started over his right groin and also involved his trunk and scalp about two months following stent placement [6]. Nickel seemed to be the etiology of allergy to stainless steel stents in that patient. Stainless steel stents contain various amounts of nickel between 9% and 57 % [6].

In a case report published in 2013, Chaudhry and colleagues described two cases of delayed hypersensitivity presenting as dermatitis following the implantation of the spinal cord stimulator devices. An allergy to polyurethane lead extensions was proposed as a potential cause [9].

The electrode of the SPRINT TM peripheral nerve stimulation (MicroLead) in this patient was made of stainless steel [7]. Pruritis and rash that started soon after the lead placement in our patient seem to be related to type I hypersensitivity. She also developed papules and pustules within the next few days after lead placement, which could represent acute generalized exanthematous pustulosis, a subclass of type IV hypersensitivity.

The patient tolerated the Curonix permanent peripheral nerve stimulation implant very well. The fact that no allergic reaction was observed despite the procedure site being covered with the same type of adhesive bandage rules out the possibility of an allergy to adhesive bands as an etiology for her reaction to temporary peripheral nerve stimulation lead placement. The new system's tissue-contacting parts of the electrode array are made of platinum-iridium (electrodes), polyurethane (insulation and tip), and no stainless steel or nickel [8]. It is unknown what percentage of the MicroLead used in temporary peripheral nerve stimulation systems is nickel-based. Given that our patient informed us about her remote nickel allergy after the lead removal, nickel allergy seems to be our case's potential cause of the allergic reaction.

Although allergic reactions to percutaneous peripheral nerve stimulation are rare, they can still cause serious problems. A thorough medical history, including medications and a list of environmental allergies, can help reduce the risk of unwanted reactions. Allergic reactions are still possible due to an unknown allergy to a substance or previous exposure, leading to sensitization during future contact.

The patient's consent was secured for publication of case details.

## Declaration of competing interest

The authors declare that they have no known competing financial interests or personal relationships that could have appeared to influence the work reported in this paper.
