# Supplementary material for: Allergic reaction to temporary percutaneous peripheral stimulation leads: A case report
Source: Interv Pain Med. 2025 Mar 8;4(1):100562. doi: 10.1016/j.inpm.2025.100562 (PMC11930406; doi:10.1016/j.inpm.2025.100562)
Supplement: Multimedia component 1 [file mmc1.pdf]

CARE
case report guidelines

CARE Checklist of information to include when writing a case report

(cc) BY-NC-ND

✓

Topic	Item	Checklist item description	Reported on Line
Title	1	The diagnosis or intervention of primary focus followed by the words "case report"	1
Key Words	2	2 to 5 key words that identify diagnoses or interventions in this case report, including "case report"	30-32
Abstract (no references)	3a	Introduction: What is unique about this case and what does it add to the scientific literature?	34-52
	3b	Main symptoms and/or important clinical findings	58-61
	3c	The main diagnoses, therapeutic interventions, and outcomes	58-72
	3d	Conclusion—What is the main "take-away" lesson(s) from this case?	115-119
Introduction	4	One or two paragraphs summarizing why this case is unique (**may include references**)	50-52
Patient Information	5a	De-identified patient specific information.	54
	5b	Primary concerns and symptoms of the patient	58-61
	5c	Medical, family, and psycho-social history including relevant genetic information	54-56
	5d	Relevant past interventions with outcomes	55,56, 58-61
Clinical Findings	6	Describe significant physical examination (PE) and important clinical findings.	65, 66
Timeline	7	Historical and current information from this episode of care organized as a timeline	N/A
Diagnostic Assessment	8a	Diagnostic testing (such as PE, laboratory testing, imaging, surveys).	N/A
	8b	Diagnostic challenges (such as access to testing, financial, or cultural)	N/A
	8c	Diagnosis (including other diagnoses considered)	58-61
	8d	Prognosis (such as staging in oncology) where applicable	N/A
Therapeutic Intervention	9a	Types of therapeutic intervention (such as pharmacologic, surgical, preventive, self-care)	56, 70, 71
	9b	Administration of therapeutic intervention (such as dosage, strength, duration)	N/A
	9c	Changes in therapeutic intervention (with rationale)	70, 71, 110
Follow-up and Outcomes	10a	Clinician and patient-assessed outcomes (if available)	72
	10b	Important follow-up diagnostic and other test results	N/A
	10c	Intervention adherence and tolerability (How was this assessed?)	N/A
	10d	Adverse and unanticipated events	58-61
Discussion	11a	A scientific discussion of the strengths AND limitations associated with this case report	74-113
	11b	Discussion of the relevant medical literature **with references**.	75-113
	11c	The scientific rationale for any conclusions (including assessment of possible causes)	111-113
	11d	The primary "take-away" lessons of this case report (without references) in a one paragraph conclusion	117-119
Patient Perspective	12	The patient should share their perspective in one to two paragraphs on the treatment(s) they received	N/A
Informed Consent	13	Did the patient give informed consent? Please provide if requested	Yes ☑ No ☐
